# Studies should fully report on variables that determine the dynamics and the temperature gradients during hypothermia

**DOI:** 10.1186/s13054-018-2145-3

**Published:** 2018-09-21

**Authors:** Horia-Nicolai Teodorescu

**Affiliations:** Laboratories for Medical Electronics and Informatics, Technical University of Iasi, Iasi, Romania

Glover et al. [1] pointed out repeatedly that limited data on cooling protocols used for treatment increase the uncertainty of the results of the analysis of hypothermia outcomes, especially when surface cooling is applied. While Glover et al. provided compelling evidence that, averaged on a large number of treatment cases, a method using intravascular (IV) catheters and those relying on surface cooling (SFC) have similar outcomes, more detailed information is needed and is not currently available to select the best procedure in terms of target temperature, duration of attaining the target temperature, period of maintaining it, temperature spatial distribution (gradients) on the skull and in the brain, speed of cooling and rewarming, and area and temperature of the cooling elements in the case of SFC, etc. Currently it is not mandatory to document the steps of the cooling process with full details. When using external cooling (pads, ice bags) with ad-hoc decision on their placement on the head, the cooling procedure is qualitatively described, which is unsuitable for deriving treatment guidance. Glover et al. used all the available variables; however, they point out, echoing the dissatisfaction of Rhodes [2] and Dietrich and Bramlett [3], that more refined data, such as precise temperature variation curves, would be useful but are typically missing.

Systematically reporting the various variables that may influence the outcome is needed for reproducibility of the hypothermia treatment. As a minimum, equivalent physical conditions must be established between treatment cases using the same cooling method (PV or SFC).

For brain hypothermia, variables that may influence the outcome include the spatial non-uniformity (gradients) of the temperature in the brain, the area of the skull actually cooled, and the temperature of the cooling pads. Minamisawa et al. [4] stressed that different parts of the brain are differently protected by hypothermia. This variability may be an issue when thermal gradients are present across those regions. This connects with the potentially higher gradients in the brain in the case of externally applied cooling devices than those with IV. Furthermore, the uncertainty regarding the achieved temperature, the duration of cooling, and the thermal gradients in the brain is higher when bags with ice are applied. All these are strong reasons not to include cases treated with external ice bags and cases where the area of the cooling is not reported in studies on the effects of brain hypothermia. Moreover, a tight standardization of the SFC procedures is highly desirable.
